# Systemic immune inflammation index and system inflammation response index are potential biomarkers of atrial fibrillation among the patients presenting with ischemic stroke

**DOI:** 10.1186/s40001-022-00733-9

**Published:** 2022-07-02

**Authors:** Kai-bin Lin, Feng-hua Fan, Ming-qi Cai, Yin Yu, Chuan-liang Fu, Lu-yue Ding, Yu-dong Sun, Jia-wen Sun, Yong-wang Shi, Zhi-feng Dong, Min-Jie Yuan, Shuai Li, Yan-peng Wang, Kan-kai Chen, Ji-ni Zhu, Xin-wei Guo, Xue Zhang, Yu-wu Zhao, Jing-bo Li, Dong Huang

**Affiliations:** 1 Heart Center, Shanghai Jiaotong University Affiliated Sixth People’s Hospital, School of Medicine, Shanghai Jiaotong University, Xuhui District, No. 600, Yishan Road, Shanghai, People’s Republic of China; 2 Department of Cardiology, Shanghai Institute of Cardiovascular Diseases, Zhongshan Hospital, Fudan University, Shanghai, People’s Republic of China; 3 Department of Cardiology, Shanghai East Hospital, School of Medicine, Tongji University, Shanghai, People’s Republic of China; 4 School of Medicine, Shanghai Jiaotong University, Shanghai, People’s Republic of China; 5 School of Biomedical Engineering, Shanghai Jiao Tong University, Shanghai, People’s Republic of China; 6 Zhiyuan College, Shanghai Jiaotong University, Shanghai, People’s Republic of China; 7 Department of Neurology, Shanghai Jiao Tong University Affiliated Sixth People’s Hospital, School of Medicine, Shanghai Jiaotong University, Shanghai, People’s Republic of China

**Keywords:** Atrial fibrillation, Systemic immune inflammation index, Systemic inflammation response index, Ischemic stroke

## Abstract

**Background:**

Chronic inflammatory disorders in atrial fibrillation (AF) contribute to the onset of ischemic stroke. Systemic immune inflammation index (SIII) and system inflammation response index (SIRI) are the two novel and convenient measurements that are positively associated with body inflammation. However, little is known regarding the association between SIII/SIRI with the presence of AF among the patients with ischemic stroke.

**Methods:**

A total of 526 ischemic stroke patients (173 with AF and 353 without AF) were consecutively enrolled in our study from January 2017 to June 2019. SIII and SIRI were measured in both groups. Logistic regression analysis was used to analyse the potential association between SIII/SIRI and the presence of AF. Finally, the correlation between hospitalization expenses, changes in the National Institutes of Health Stroke Scale (NIHSS) scores and SIII/SIRI values were measured.

**Results:**

In patients with ischemic stroke, SIII and SIRI values were significantly higher in AF patients than in non-AF patients (all *p* < 0.001). Moreover, with increasing quartiles of SIII and SIRI in all patients, the proportion of patients with AF was higher than that of non-AF patients gradually. Logistic regression analyses demonstrated that log-transformed SIII and log-transformed SIRI were independently associated with the presence of AF in patients with ischemic stroke (log-transformed SIII: odds ratio [OR]: 1.047, 95% confidence interval CI = 0.322–1.105, *p* = 0.047; log-transformed SIRI: OR: 6.197, 95% CI = 2.196–17.484, *p* = 0.001). Finally, a positive correlation between hospitalization expenses, changes in the NIHSS scores and SIII/SIRI were found, which were more significant in patients with AF (all *p* < 0.05).

**Conclusions:**

Our study suggests SIII and SIRI are convenient and effective measurements for predicting the presence of AF in patients with ischemic stroke. Moreover, they were correlated with increased financial burden and poor short-term prognosis in AF patients presenting with ischemic stroke.

## Introduction

Atrial fibrillation (AF), a common type of arrhythmia, affects 14% of the adult population and is largely diagnosed in the elderly [1]. AF-related stroke accounts for more than 79% of all cardiogenic strokes, and its risk increases with a higher AF burden [2]. Furthermore, AF-related stroke usually results in worse outcomes and greater expenses than non-AF stroke [3]. Thus, monitoring the cardiac rhythm of stroke patients is an important strategy in clinical practice [4]. Traditionally, the cost of multiple cardiac rhythm investigations increases substantially when traditional detection methods of AF in stroke patients fails, doubling from 5.3% to 10.9% [5]. In recent years, biomarkers, which can predict AF in ischemic stroke patients, have been developed and investigated. Inflammatory activity has been shown to be related to AF and its complications [6]. Certain inflammatory markers, such as C-reactive protein and interleukin-6, have shown that a strong relationship exists between AF burden and poor sinus rhythm maintenance [7, 8]. However, inflammatory biomarkers for assessing AF-related stroke have not been studied in detail.

Inflammatory activity can be evaluated by a series of haematological indices derived from white blood cells (WBC) and its elements. The neutrophil-to-lymphocyte ratio and platelet-to-lymphocyte ratios have demonstrated an enhanced predictive potential in the prognosis of cardiovascular diseases (CVDs) and related mortality [9–11].

Recently, two novel inflammatory markers, systemic immune inflammation index (SIII) and system inflammation response index (SIRI), were able to provide additional information in the risk assessment of CVDs [12]. This study aimed to further determine the associations between SIII and SIRI with AF among the stroke patients.

## Methods

### Study design

#### Study population

**Fig. 1:**
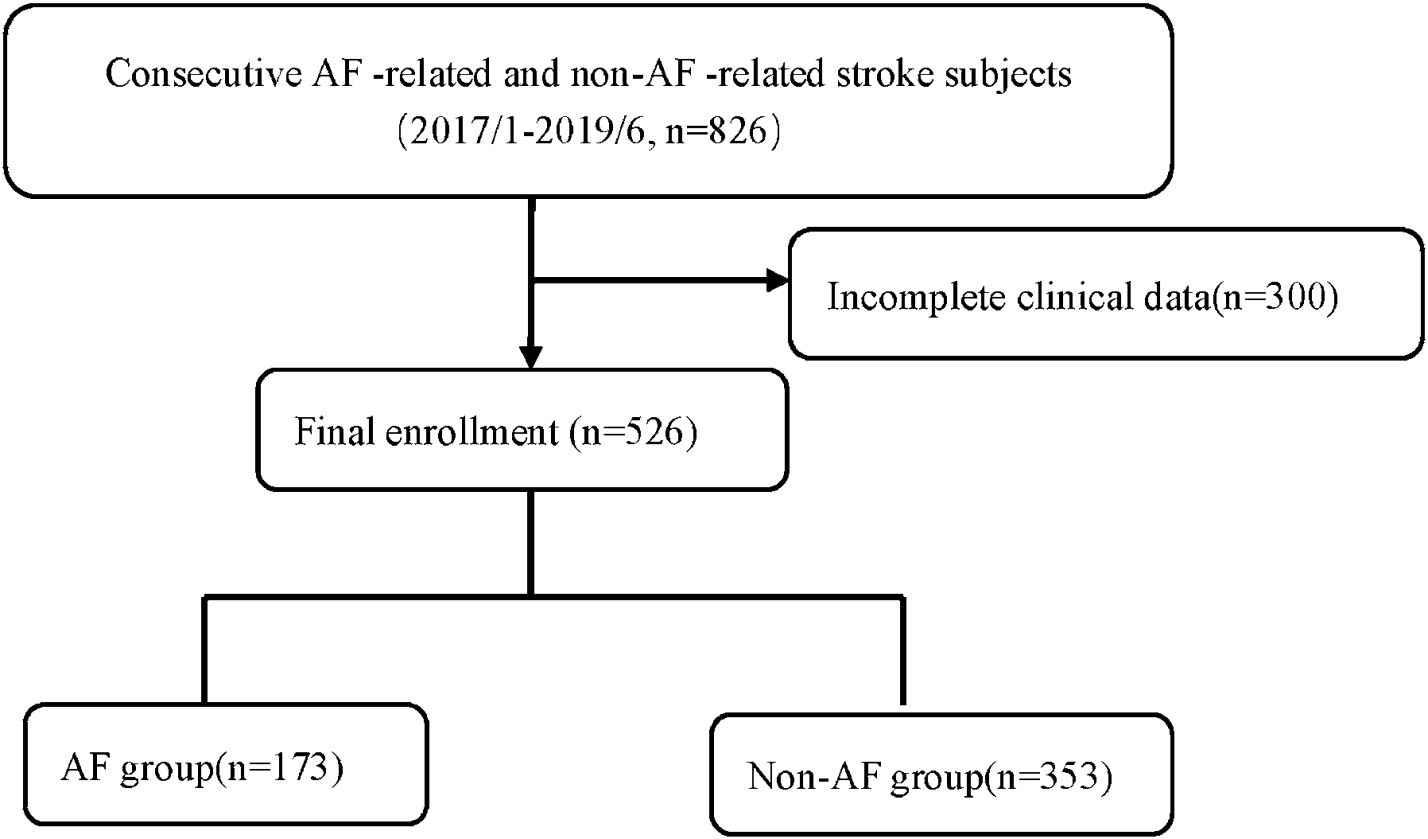
Flow chart of patient enrolment in our study. AF, atrial fibrillation

This study was conducted from January 2017 to June 2019. We collected information on 826 eligible hospitalized patients with a diagnosis of acute ischemic stroke who underwent brain magnetic resonance imaging. Furthermore, computed tomography, transthoracic echocardiography, electrocardiography (ECG), and Holter were carried out to elucidate stroke mechanisms. Based on these results, patients were classified using the Trial of Org 10172 in Acute Stroke Treatment (TOAST) criteria into cardioembolism, atherosclerosis, lacunar, and other determined and undetermined causes. AF-related stroke was defined as stroke in patients with either a history of AF or clinical evidence of AF based on ECG/24-h Holter monitoring during hospitalization [13]. The exclusion criteria were presence of malignant tumours and haematological, autoimmune, inflammation-related or chronic liver/kidney diseases. Finally, 526 patients with complete clinical data were included in the final analysis (Fig. 1).

#### Clinical and biochemical assessments

Blood samples were obtained from all patients during hospitalization after fasting for at least 12 h. Levels of total cholesterol (TC), triglycerides (TG), low-density lipoprotein-cholesterol (LDL-C) and high-density lipoprotein-cholesterol (HDL-C) were assessed using a HITACHI 912 analyser (Roche Diagnostics, Germany). Routine blood tests, including platelet, neutrophil, lymphocyte, and monocyte counts, were performed using a full blood count analyser (Sysmex XT-1800i, Sysmex Corporation).

Detailed information about the patients’ medical histories (hypertension, diabetes and coronary artery disease) and lifestyles (smoking) were obtained using a standard questionnaire. The National Institutes of Health Stroke Scale score on admission (pre-NIHSS) and discharge (discharge-NIHSS) were recorded to assess neurological function. The financial burden was evaluated based on the cost of hospitalization per day and the total charges upon discharge.

Echocardiography was performed during hospitalization by trained sonologists. Meanwhile, information regarding the infarcted and ischemic areas of the brain was analysed by experienced radiologists.

#### SIII and SIRI measurement

SIII was calculated as follows: peripheral platelet count × neutrophil count/lymphocyte count. SIRI was calculated as follows: neutrophil count × monocyte count/lymphocyte count.

### Statistical analysis

For baseline characteristics, the Kolmogorov–Smirnov test was used to test the normality of the distribution. Continuous variables of normal distribution are expressed as ± standard deviation, and differences among groups were analysed by the Student’s *t* test or one-way analysis of variance. Medians (Q1, Q3) are summarized for non-normally distributed variables. Logarithmic transformations were calculated for the non-normally distributed continuous variables (SIII and SIRI). Analysis of covariance was used to evaluate logarithmic transformations of SIII and SIRI between two groups while considering age, sex, history of hypertension, diabetes, and history of stroke/transient ischemic attack (TIA) as covariates. Logistic regression models were used to analyse the potential association of SIII and SIRI with the presence of AF when age, sex, history of history of hypertension, diabetes and history of stroke/TIA were considered. Correlations between variables were analysed using Pearson's correlation test. Parameters associated with SIII and SIRI were identified using multivariate linear regression analysis. Statistical analyses were performed using SPSS 25.0 (SPSS, Inc., Chicago, IL, USA). Statistical significance was defined as a two-tailed *P* < 0.05.

## Results

### Characteristics of the studied population

A total of 173 AF-related stroke and 353 non-AF related stroke patients, respectively, were analysed in our study (Table 1). Compared to non-AF patients, AF patients were younger and had a lower prevalence of diabetes mellitus and cigarette use. Moreover, there was a lower proportion of male patients among those with AF. In our study, the patients who were diagnosed with AF-related cardiogenic stroke did not suffer from rheumatic valve diseases, left ventricular thrombus, or congenital heart disease. Among non-AF patients, atherosclerosis was the most frequent stroke subtype (*n* = 222, 62.8%) followed by small vessel disease (*n* = 127, 35.9%), while other causes accounted for 1.1% (*n* = 4). The distribution of the infarcted area also suggested a significant difference between the two groups. A larger left atrium diameter was observed in AF patients, which indicated more serious left atrial remodelling. In addition, the CHA2DS2–VASc score of the AF group was 4.90 ± 1.37.

**Table 1:** Baseline characteristics of patients with atrial fibrillation (AF)-related and non-AF-related stroke

	No AF (*n* = 353)	Definite AF (*n* = 173)	*p* value
Age, years	78.61 ± 9.65	68.08 ± 12.16	< 0.001
BMI (kg/m^2^)	23.51 ± 3.87	24.20 ± 3.11	0.046
Male, *n* (%)	210 (59.49%)	67 (38.72%)	< 0.001
Hypertension, *n* (%)	297 (84.13%)	143 (82.65%)	0.677
Diabetes mellitus, *n* (%)	122 (34.56%)	32 (18.49%)	< 0.001
Coronary artery disease, *n* (%)	37 (10.48%)	29 (16.76%)	< 0.001
Current cigarette use, n (%)	59 (16.71%)	16 (9.24%)	0.002
Previous stroke/TIA, *n* (%)	109 (30.87%)	63 (36.41%)	0.262
Infarction area			
Anterior circulation area	55 (15.58%)	46 (26.58%)	< 0.001
Partial anterior circulation area	181 (51.27%)	81 (46.82%)	
Posterior circulation area	90 (25.49%)	24 (13.87%)	
Multiple lacunar infarction	23 (6.51%)	19 (11.00%)	
CHA_2_DS_2_–VASc score	NA	4.90 ± 1.37	
Treatment methods			
Intravenous thrombolysis	28 (7.93%)	27 (15.61%)	< 0.001
Interventional therapy	13 (3.68%)	25 (14.45%)	
None	312 (88.39%)	121 (69.94%)	
NIHSS (admission)	3 (1, 6)	10 (3, 18)	< 0.001
NIHSS (discharge)	2 (1, 4)	5 (2, 12)	< 0.001
Cost per day (RMB)	1631.37 ± 438.38	1,885.50 ± 614.43	< 0.001
LAd, mm	36.69±4.98	43.23±6.93	< 0.001
LVDd, mm	44.58 ± 8.58	45.92 ± 6.33	0.174
TC (mmol/L)	4.68 ± 1.01	4.32 ± 1.22	0.002
TG (mmol/L)	1.63 ± 0.62	1.17 ± 1.05	< 0.001
LDL-C (mmol/L)	2.88 ± 0.82	2.49 ± 0.98	< 0.001
HDL-C (mmol/L)	1.07 ± 0.35	1.27 ± 0.29	< 0.001
hs-CRP (mg/L)	2.7 (0.9, 5.4)	3.3 (1.0, 5.9)	0.057
White blood cell count, × 10^9^/L	8.50 ± 3.86	7.73 ± 2.65	0.007
Neutrophils, × 10^9^/L	6.38 ± 3.78	5.37 ± 2.62	0.002
Lymphocytes, × 10^9^/L	1.40 ± 0.75	1.72 ± 0.68	< 0.001
Monocytes, × 10^9^/L	0.54 ± 0.24	0.49 ± 0.28	0.051
Platelet count, × 10^9^/L	198.00 ± 67.46	214.66 ± 66.56	0.008
SIII	562.50 (379.73–1,040.33)	802.29 (473.08–1,390.30)	< 0.001
SIRI	1.28 (0.78–2.12)	2.05 (1.17–4.02)	< 0.001

CHA_2_DS_2_–VASc, congestive heart failure, hypertension, age ≥ 75 years, diabetes mellitus, TIA, vascular disease, age 65–74 years, sex category

*BMI* body mass index, *TIA*, transient ischemic attack, *NIHSS* National Institutes of Health Stroke Scale, *LAD* left atrial diameter, *LVDd* left ventricular end diastolic dimension, *LDL*-*C* low density lipoprotein cholesterol, *HDL*-*C* high density lipoprotein cholesterol, *TC* total cholesterol, *TG* triglyceride, *SIII* systemic immune inflammation index, *SIRI* system inflammation response index, *hs*-*CRP* High sensitive C-reactive protein

Among the patients with a history of ischemic stroke in both groups, AF patients suffered more significant neurological deficits than non-AF patients (NIHSS scores at discharge:,4 (2, 8) vs. 2 (0, 5), *P* < 0.001).TC, TG and LDL-C levels were lower, whereas HDL-C levels were higher, in AF patients than in non-AF patients. Considering the total WBC, neutrophil, lymphocyte and monocyte counts, AF patients demonstrated lower levels than non-AF patients. The platelet count was elevated in AF patients, and both SIII and SIRI was significantly higher in AF patients than in non-AF patients (SIII: 562.50 [379.73, 1,040.33] vs. 802.29 [473.08, 1,390.30], *P* < 0.001; SIRI: 1.28 [0.78, 2.12] vs. 2.05 [1.17, 4.02], *P* < 0.001). Logarithmic transformations were calculated for the non-normally distributed continuous variables (SIII and SIRI). We found that the difference in log-transformed SIII and SIRI were still significant even when sex, age, hypertension, and diabetes were added as covariates (all *P* < 0.001). Meanwhile, we observed that the median high sensitive C-reactive protein level at baseline was marginally higher in AF patients than in non-AF patients (3.3 [1.0, 5.9] vs. 2.7 [0.9, 5.4] mg/L, *P* = 0.057).

### SIII and SIRI values in AF and non-AF patients

**Fig. 2:**
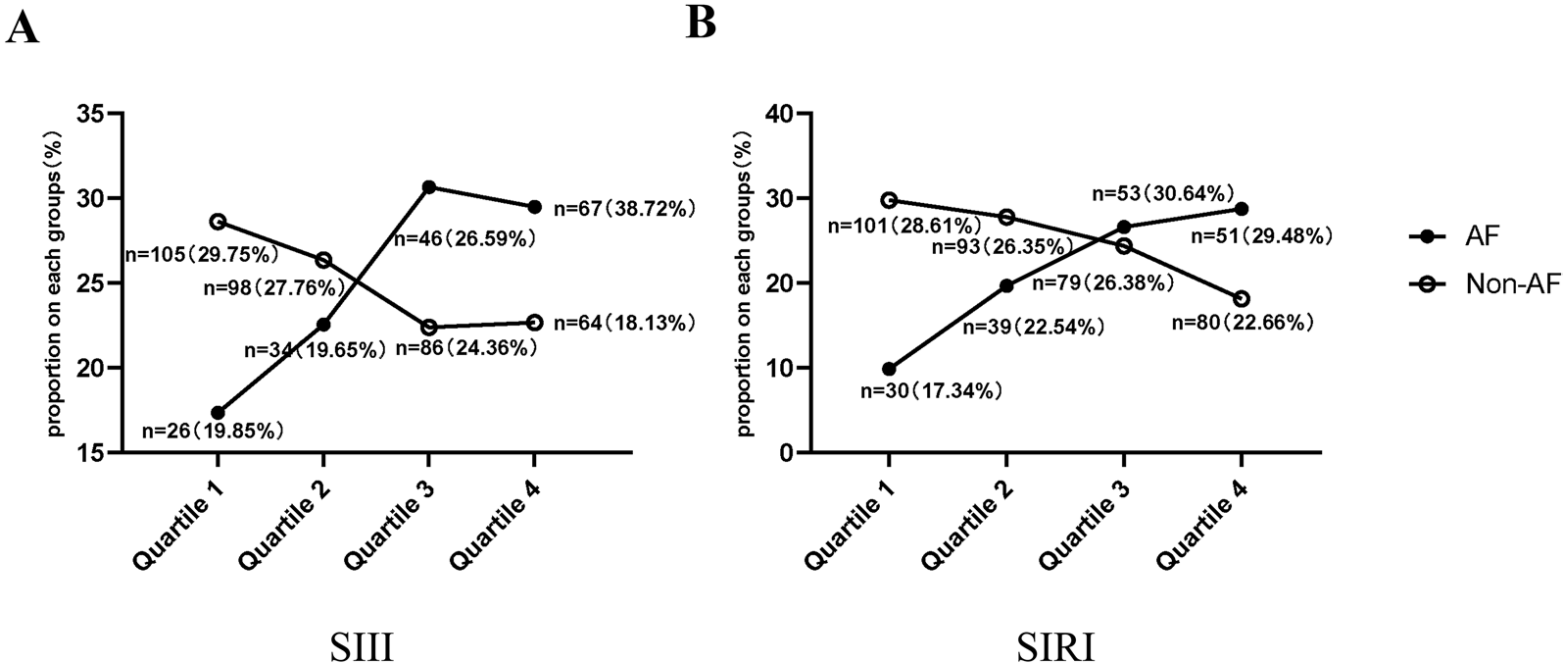
Distribution of proportions in atrial fibrillation (AF) and non-AF subgroups among different quartiles of the systemic immune inflammation index (SIII) (**A**) and system inflammation response index (SIRI) (**B**) in patients with stroke

We further described the variation in the characteristics of the proportion of each subgroup in the quartiles of SIII and SIRI in all patients (Fig. 2A, B). In both quartiles 1 and 2, the proportion of AF patients was lower than that of non-AF patients (SIII: [Q1] 19.85% vs. 29.75%; SIRI: [Q1] 17.34% vs. 28.61%; SIII: [Q2] 19.65% vs. 27.76%; SIRI: [Q2] 22.54% vs. 26.35%). However, we observed an upward trend in the proportion of AF patients with increasing quartiles of SIII and SIRI, which was contrary to that in non-AF patients. Hence, in both quartiles 3 and 4, the proportion of AF patients was higher than that of non-AF patients (SIII: [Q3] 26.59% vs. 24.36%; SIRI: [Q3] 30.64% vs. 26.38%; SIII: [Q4] 38.72% vs. 18.13%; SIRI: [Q4] 29.48% vs. 22.66%).

### Multivariate linear regression and logistic regression analysis for SIII and SIRI

Results of multivariate linear regression analysis with age, male sex, hypertension, diabetes mellitus, history of stroke/TIA and AF as covariates are presented in Tables 2 and 3. In addition to age, AF was independently associated with elevated log-transformed SIII (*P* = 0.026) and log-transformed SIRI (*P* < 0.001). Logistic regression analysis revealed that log-transformed SIII and log-transformed SIRI were independently associated with an increased risk of AF in patients with stroke. (log-transformed SIII: OR: 1.047, 95% CI = 0.322–1.105, *P* = 0.047; log-transformed SIRI: OR: 6.197, 95% CI = 2.196–17.484, *P* = 0.001, Table 4).

**Table 2:** Multivariate linear regression analysis for log systemic immune inflammation index (SIII)

	95% CI	Sβ	*p *value
Age	0.003 (0.00–0.006)	0.102	0.036
Male	0.012(− 0.073–0.050)	0.016	0.714
Hypertension	0.014(− 0.097–0.069)	0.015	0.735
Diabetes	0.013(− 0.050–0.032)	0.023	0.474
Stroke history	0.025(− 0.083–0.037)	0.033	0.402
AF	0.082 (0.010–0.155)	0.109	0.026

*CI* confidence interval, *Sβ* standard error of regression coefficient, *AF* atrial fibrillation

**Table 3:** Multivariate linear regression analysis for log system inflammation response index (SIRI)

	95% CI	Sβ	*p* value
Age	0.004 (0.001–0.007)	0.132	0.006
Male	0.025 (− 0.043–0.093)	0.032	0.467
Hypertension	0.016 (− 0.107–0.75)	0.015	0.734
Diabetes	0.006 (− 0.046–0.035)	0.012	0.783
Stroke/TIA history	0.016 (− 0.080–0.048)	0.021	0.632
AF	0.149 (0.069–0.228)	0.176	< 0.001

*CI* confidence interval, *Sβ* standard error of regression coefficient, *TIA* transient ischemic attack, *AF* atrial fibrillation

**Table 4:** Logistic regression analysis for log systemic immune inflammation index (SIII) and log system inflammation response index (SIRI) in patients with and without atrial fibrillation (AF)

	OR	95% CI	*p* value
Age	1.087	1.063–1.111	< 0.001
Male	1.838	1.189–2.842	0.006
Hypertension	1.267	0.707–2.269	0.427
Diabetes mellitus	1.237	0.651–2.352	< 0.001
Stroke /TIA history	1.871	0.508–6.893	0.346
log SIII	1.047	0.322–1.105	0.047
log SIRI	6.197	2.196–17.484	0.001

. After adjustment for age, sex, hypertension, diabetes and stroke/TIA history

*CI* confidence interval, *TIA* transient ischemic attack

### Correlation between financial burden/changes in NIHSS Scores and SIII/SIRI

**Fig. 3:**
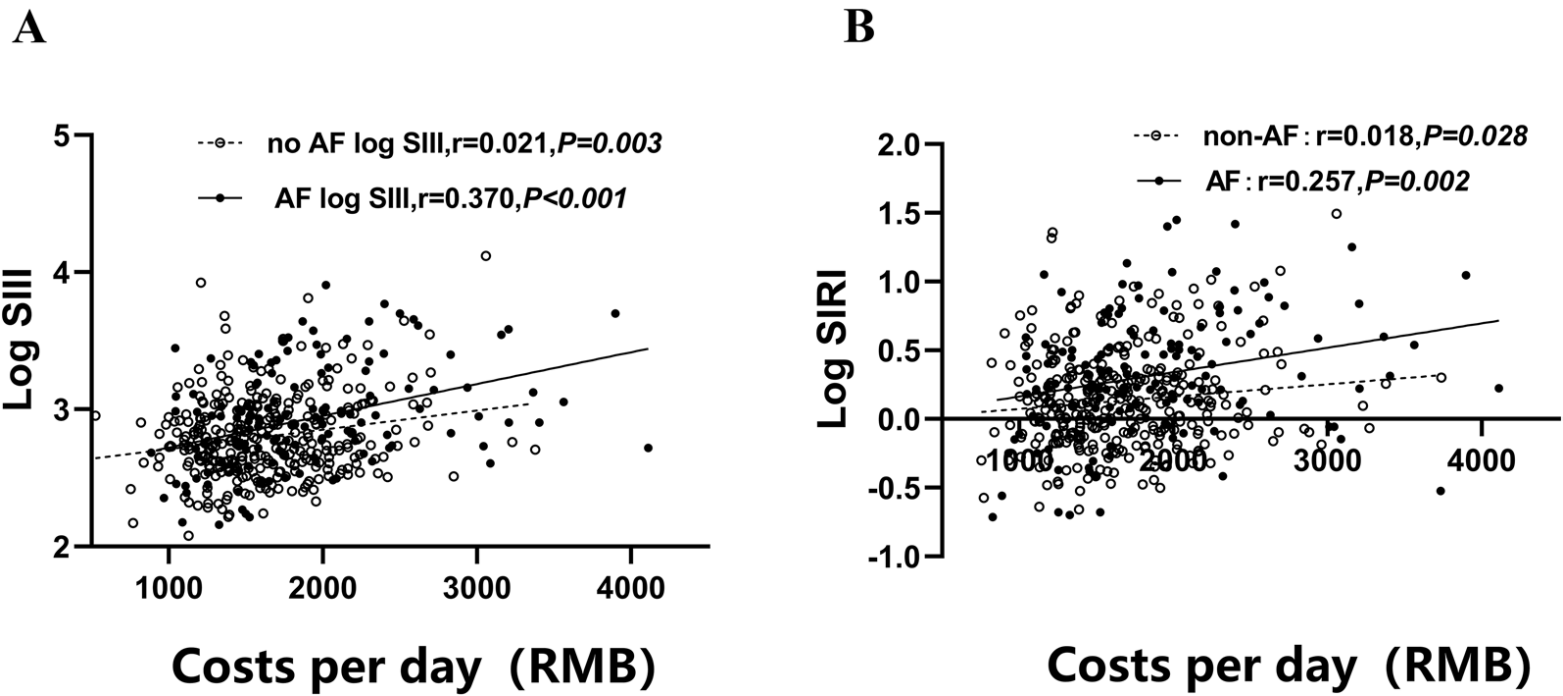
Correlation between costs and systemic immune inflammation index (SIII) (or system inflammation response index [SIRI]). SIII and SIRI were logarithmically transformed before plotting. Hollow circles and dotted lines, non-atrial fibrillation (AF) patients (*n* = 353); Filled circles and solid lines, patients with AF (*n* = 173)

Compared with non-AF related ischemic stroke, AF-related stroke resulted in a higher disability and mortality rate, which led to greater financial burden to society [14]. Then we determined the potential of SIII and SIRI for evaluating the financial burden of AF in the patients with stroke. In our study, the daily expenses of AF-related stroke patients were significantly higher than that non-AF stroke patients (1885.50 ± 614.43 vs. 1631.37 ± 438.38 RMB, *P* < 0.001). Moreover, as shown in Fig. 3A, B, we found that the daily expenses during hospitalization correlated significantly with log-transformed SIII and SIRI. Moreover, AF-related stroke patients exhibited a larger correlation coefficient compared with non-AF stroke patients (SIII: r = 0.370, *p* < 0.001 vs. r = 0.021, *p* = 0.003; SIRI: r = 0.257, *p* = 0.002 vs. r = 0.018, *p* = 0.028).

**Fig. 4:**
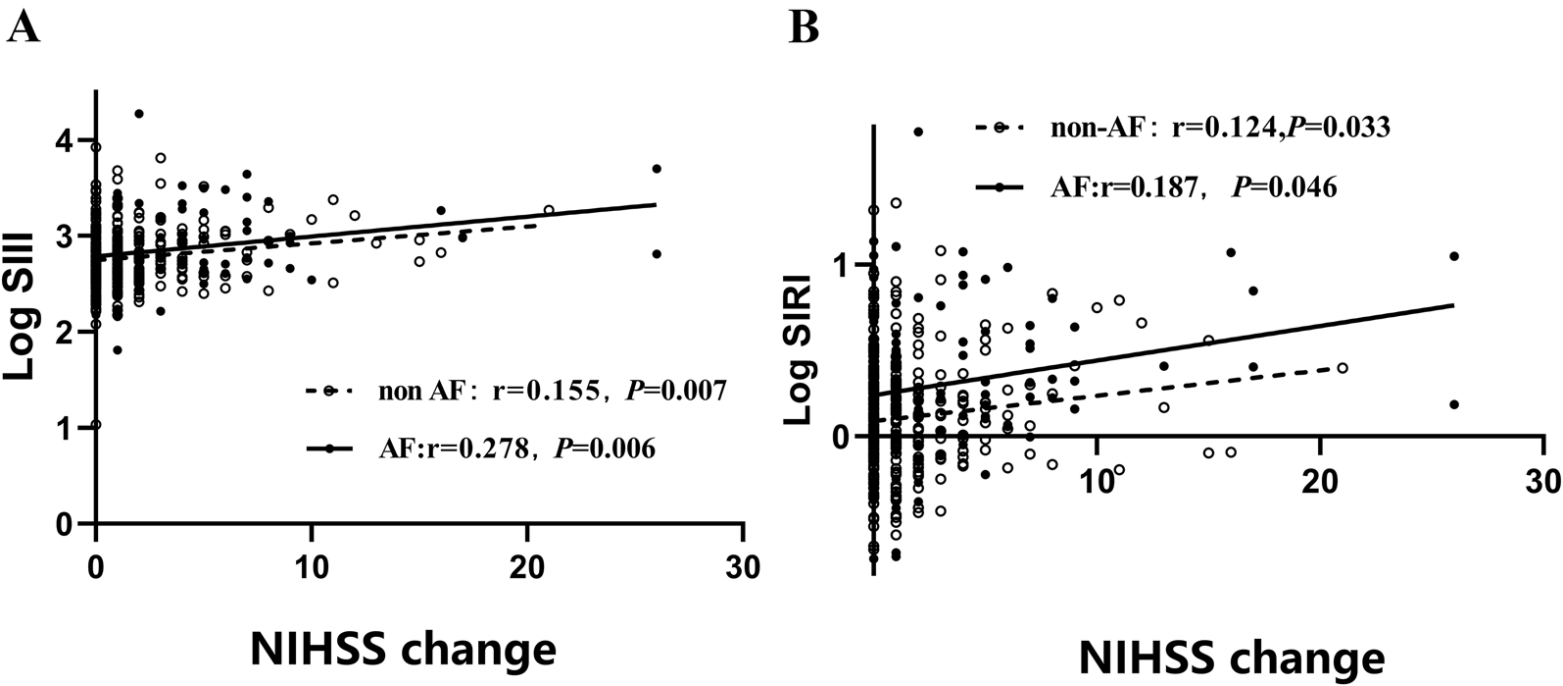
Correlation between change in National Institutes of Health Stroke Scale (NIHSS) scores and systemic immune inflammation index (SIII) (or system inflammation response index [SIRI]). SIII and SIRI were logarithmically transformed before plotting. Hollow circles and dotted lines, non-atrial fibrillation (AF) patients (*n* = 353); Filled circles and solid lines, patients with AF (*n* = 173)

Although the proportion of AF patients with stroke receiving intravenous thrombolysis/interventional therapy was higher than that of non-AF stroke patients, AF patients had higher NIHSS scores on admission and discharge (all *P* < 0.05). We also observed that changes in the NIHSS scores of AF patients had a larger correlation coefficient with the log-transformed SIII and SIRI than non-AF patients (SIII: r = 0.278, *P* = 0.006 vs. r = 0.155, *P* = 0.007; SIRI: r = 0.187, *P* = 0.046 vs. r = 0.124, *P* = 0.033) (Fig. 4A, B).

## Discussion

In this study, we investigated the association between SIII, SIRI and AF-related stroke. The results demonstrated that SIII and SIRI values were significantly higher in AF group than in non-AF group among the stroke patients. Meanwhile, SIRI and SIII were both independently associated with the presence of AF in patients with ischemic stroke. Moreover, the positive correlation between SIII, SIRI, hospitalization expenses and changes in NIHSS scores were enhanced in AF patients than in non-AF patients in the patients with ischemic stroke.

Regardless of the type of stroke, the immune response to acute cerebral ischemia is a major factor in stroke pathobiology and prognosis [15]. Patients with AF demonstrate a phenomenon of systemic inflammation, which will exacerbate the inflammatory injury of stroke [16]. Moreover, there is plausible evidence linking inflammation to the initiation of AF-related thrombosis. Endothelial activation/damage, increased platelet activation and increased fibrinogen expression contribute to AF-related stroke [17]. Thus, the identification of sensitive and specific inflammatory biomarkers for AF among the stroke patients further guides individualized therapy and achieves the goal of precision medicine [18]. SIII and SIRI can reflect the systemic inflammatory activity of the body, and they have great potential in predicting the prognosis of cancer patients [19–21]. Higher monocyte and neutrophil counts and lower lymphocyte counts have been associated with a higher CVD risk [22–24]. Based on previous research, SIII and SIRI have been proven to be related to the risk of CVDs and all-cause death. More importantly, higher SIII and SIRI values correlated with an increased risk of stroke, stroke subtypes (ischemic and haemorrhagic stroke) and all-cause death [12]. However, whether the absence and presence of AF influences the level of SIII and SIRI in ischemic stroke merits further investigation.

A high neutrophil-to-lymphocyte ratio is more closely related to new-onset AF and recurrence after AF ablation [25]. Elevated monocyte counts also result in the development of atrial remodelling [26]. Although WBC, neutrophil, lymphocyte, and monocyte counts were significantly lower in AF-related stroke patients than in non-AF stroke patients in our study, AF patients had higher SIRI and SIII, which demonstrated that the onset of AF-related stroke is a systemic inflammatory process. Cerebrovascular occlusion initiates a local inflammatory immune response and post-stroke immunosuppression [27]. The absolute value of the WBC count could suggest that post-stroke immunosuppression is more significant in patients with AF. In addition, there is excess platelet activation and increased platelet aggregation in patients with AF, which was deduced by higher levels of CD62P, CD63 and sP-selectin [28]. Platelet disorder is associated with an inflammatory response [29]. In addition, the higher SIRI/SIII values in AF patients could also confirm previous findings.

Our data revealed that SIRI and SIII were both independently associated with the presence of AF in patients with stroke. Changes in NIHSS scores can be used to assess the improvement of neurological function in stroke patients. The positive correlation between the change in NIHSS scores and SIII /SIRI was markedly enhanced in patients with AF-related stroke comparing with patients with non-AF stroke. In addition, the positive correlation between hospitalization costs and SIII/ SIRI also reflected the same trend.

Screening of convenient and effective biomarkers associated with AF among the ischemic stroke patients was performed to determine the mechanisms and prognosis of AF [30]. Our data emphasized the role of inflammatory activity in the presence of AF in stroke patients.

Our study has several limitations. First, this study was a retrospective analysis based on prospectively collected data, and all enrolled patients were from a single centre. Second, the sample size in our study was modest; a large-scale prospective study is necessary to confirm our conclusions. Third, the SIII and SIRI were measured only once, which may not show dynamic changes during emergency admission. Dynamically monitoring the two indices may provide more information. Finally, the predictive ability of SIII and SIRI in the prognosis of different stroke subtypes is worth investigating.

In conclusion, this study indicated that elevated SIII and SIRI values are potential biomarkers of AF among the ischemic stroke patients.

## Data Availability

The data presented in this study are available on request from the corresponding author.
